# Artificial Mercosur license plates dataset

**DOI:** 10.1016/j.dib.2020.106554

**Published:** 2020-12-05

**Authors:** Gilles Velleneuve Trindade Silvano, Ivanovitch Silva, Vinícius Campos Tinoco Ribeiro, Vitor Rodrigues Greati, Aguinaldo Bezerra, Patrícia Takako Endo, Theo Lynn

**Affiliations:** a Universidade Federal do Rio Grande do Norte (UFRN), Rio Grande do Norte, Brazil; b Universidade de Pernambuco (UPE), Pernambuco, Brazil; c Irish Institute of Digital Business, Dublin City University, Dublin, Ireland

**Keywords:** License plates images, Mercosur license plates, Automated license plate recognition license, Plate detection, Number Plate Detection, Smart Cities, Deep Learning, Synthetic Data

## Abstract

Mercosur (a.k.a. Mercosul) is a trade bloc comprising five South American countries. In 2018, a unified Mercosur license plate model was rolled out. Access to large volumes of ground truth Mercosur license plates with sufficient presentation variety is a significant challenge for training supervised models for license plate detection (LPD) in automatic license plate recognition (ALPR) systems. To address this problem, a Mercosur license plate generator was developed to generate artificial license plate images meeting the new standard with sufficient variety for ALPR training purposes. This includes images with variation due to occlusions and environmental conditions. An embedded system was developed for detecting legacy license plates in images of real scenarios and overwriting these with artificially generated Mercosur license plates. This data set comprises 3,829 images of vehicles with synthetic license plates that meet the new Mercosur standard in real scenarios, and equivalent number of text files containing label information for the images, all organized in a CSV file with compiled image file paths and associated labels.

## Specifications Table

**Table:** 

Subject	Computer Vision and Pattern Recognition.
Specific subject area	Automatic License Plate Recognition (ALPR) with synthetic generation and embedding of Mercosur License Plates.
Type of data	Table Image Labels
How data were acquired	This data set is composed of images of vehicles, located in real scenarios, featuring artificially-generated license plates that are compliant with the new Brazilian Mercosur standard. The images are obtained from an ALPR process [1] built to detect legacy three-letter license plates in original images from a base set, that were subsequently replaced by artificially-generated Mercosur license plates, preserving the original place and slope. The base set images were ac- quired from five different sources: (1) a public traffic monitoring camera video stream at 800 × 600 resolution. (2) a digital camera at 6 Megapixels resolution, (3) zoomed and cropped versions of selected images of (2) 1, (4) a Samsung Galaxy Tab 10.1 tablet camera at 8 Megapixels resolution, and (5) an Asus ZenFone 5 smartphone camera at 12 Megapixels resolution.
Data format	Raw Analyzed Filtered
Parameters for data collection	The different sources used to build the base set resulted in a diverse amount of visible license plates, slope, resolution, shadows, occlusion and other environ- mental conditions. The images must have at least one legacy three-letter license plate detected by the ALPR system.
Description of data collection	An ALPR procedure is applied to the base set of images, resulting in a new data set in which original license plates (the legacy license plate standard) are overwritten by artificially-generated ones based on the new Brazilian Mercosur standard.
Data source location	The data was acquired in Brazi from a parking lot at IMD/UFRN - Instituto Metrópole Digital, Natal, Rio Grande do Norte and from traffic monitoring cam- eras of Ave. Protásio Alves, Porto Alegre, Rio Grande do Sul.
Data accessibility	The data set is in a Mendeley public repository available online at https://data.mendeley.com/datasets/nx9xbs4rgx.
Data ownership	The data set was generated and is under possession of the authors of this paper
Related research article	V. Ribeiro et al., "Brazilian Mercosur License Plate Detection: A Deep Learning Approach Relying on Synthetic Imagery," 2019 IX Brazilian Symposium on Computing Systems Engineering (SBESC), Natal, Brazil, 2019, pp. 1–8, doi:10.1109/SBESC49506.2019.9046091

## Value of the Data

• This data set is one of the few open access Mercosur license plate data sets and applies to new license plates issued in Brazil, Argentina, Brazil, Paraguay, and potentially Venezuela in the future.
• The data set features Mercosur license plates in a variety of realistic and naturalistic settings including both moving and stationary environments, and featuring a wide range of presentation variety including slopes, shadows and occlusions.
• This data set can be used by research institutions, manufacturers and independent software vendors in the development and testing of LPD, ALPR and other systems involving license plate detection.

## 1 Data Description

The legacy Brazilian license plate standard was introduced in 1998 and is characterized by three-letter and four-digit sequence above which are the state code and municipality. Following an agreement in 2010, the four active countries in the Mercosur trade bloc - Argentina, Brazil, Paraguay and Uruguay - agreed to roll out a unified license plate model by 2020. This data set contains images of real-life contexts where legacy three-letter license plates were detected using Tiny-YOLOv3 [2] and replaced with artificially-generated images of license plates designed to the new Mercosur standard. It is organized in two folders:

• Images – containing the image files (JPEG) of the data set; and
• Labels – containing text files with the image category identification number and the coordinates of the detected license plates in the image according to the Yolo_mark annotation specification (accessible though https://github.com/AlexeyAB/darknet#how-to-train-to-detect-your-custom-objects).

The images are organised in five categories based on their acquisition method. These are identified by a prefix in the filename:

1 monitoring_system_ – 2925 JPEG images with resolution of 800 × 600 obtained from a license plate detection model from a public traffic monitoring camera video stream at 800 × 600 resolution;
2 parking_lot1_ – 566 JPEG images with resolutions of 3264 × 2448 and 3264 × 1836 obtained using a digital camera at 6 megapixels resolution from a parking lot;
3 cropped_parking_lot_ – 315 JPEG images with diverse resolutions because this subset is composed by zoomed and cropped versions of selected images from parking_lot1_;
4 parking_lot2_ – 23 JPEG images with resolutions of 3264 × 2448 and 3264 × 1836 obtained using a Samsung Galaxy Tab 10.1 tablet camera at 8 megapixels resolution from a parking lot; and
5 parking_lot3_ – 11 JPEG images with resolutions of 3264 × 2448 and 3264 × 1836 obtained using an Asus ZenFone 5 smartphone camera from a parking lot.

In addition, the data set contains a CSV file listing all license plates featured in all images organized in seven features: *image, label, class, x_center, y_center, width* and *height*. These are further defined in Table 1.

**Table 1:** Features of the data set.

Feature	Description
Image	The name of the image file containing at least one synthetic Mercosur license plate.
Label	A text file containing the object class identifier and the coordinates of the boundary box in the image.
Class	Integer that identifies the class of the object (always zero in this project because there is only one class of object to detect).
X_center	Float number between (0 to 1] providing the coordinate *x* of the center of the boundary box.
Y_center	Float number between (0 to 1] providing the coordinate *y* of the center of the boundary box.
Width	Float number between (0 to 1] providing the coordinate *width* of the center of the boundary box.
Height	Float number between (0 to 1] providing the coordinate *height* of the center of the boundary box.

## 2 Experimental Design, Materials and Methods

**Fig. 1:**
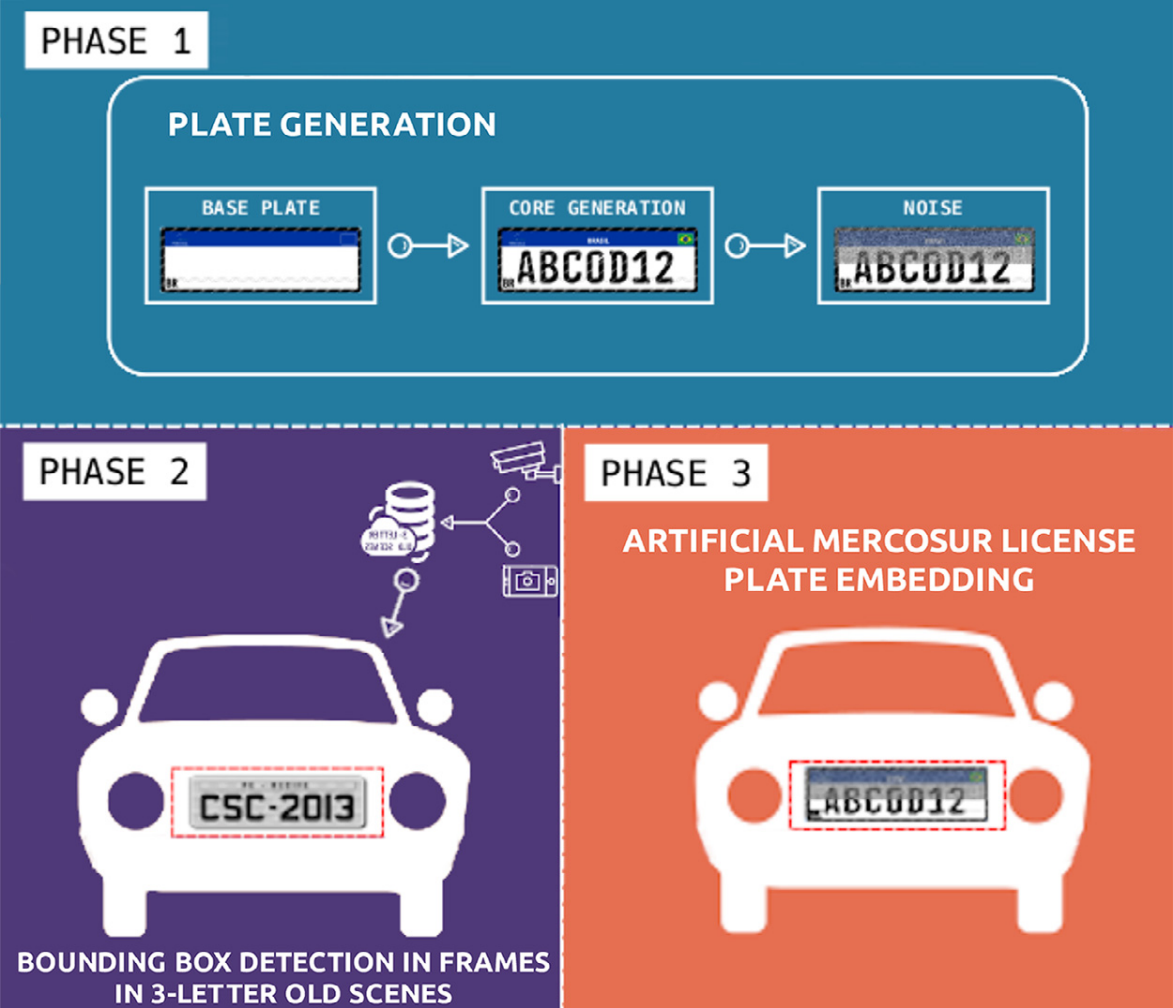
Overview of the proposed methodology.

To generate the data set, images were acquired from the sources above and then (i) coupled (a) synthetically-generated images of the new Mercosur license plates with (b) frames of real scenes containing the legacy, in this case, Brazilian, three-letter license plates, and (ii) transformed these images using various digital image processing techniques. This process is outlined in Fig. 1). The data set is a product of the first three phases of the full LPR pipeline, described in [1]. The first phase involved a number of steps. First, a Brazilian Mercosur License Plate template is created using HTML and CSS3 in accordance with the Mercosur License Plate specification. Then, a program written in *C*++ using OpenCV [3] is used to embed and position the Brazilian National flag in the template image, merge back- ground text containing required diagonal text, and generate randomly vehicle identification alphanumeric characters and write these in the template, generating a synthetic license plate.

**Fig. 2:**
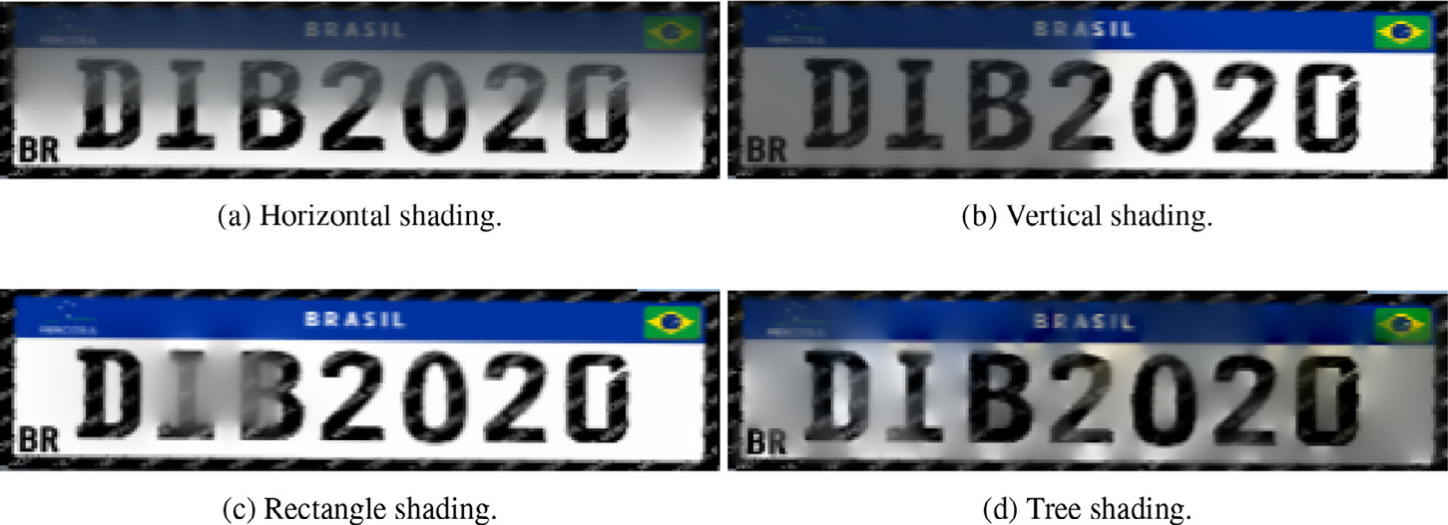
Types of artificial shading.

Once a base synthetic license plate is generated. The same *C*++ and OpenCV program apply one of four shading effects - horizontal, vertical, or rectangle and tree - randomly chosen by 1, 2, 3 and 4, respectively. The produced mask is then submitted to an appropriate non-linear transformation to its gradient field and then integrated back in the license plate with a Poisson solver. This solver locally modifies the apparent illumination of the image [4] so that the synthetic license plates integrate commonly experience shadow effects resulting from local light conditions (Fig. 2).

• **Horizontal**: This filter simulates a horizontal shadow on license plates. Thus, y_0_ and y_1_ are randomly selected in the interval [0, *w*[, and for each ***M****_ij_*, the function *h(j, y_0_, y_1_)* as presented by Equation 1.
  (1) $$h\left(j,y_{0},y_{1}\right)=\left\{ \begin{array}{cc} 255 & if\;y_{0}\leq j\leq y_{1}\\ 0 & \text{otherwise} \end{array}\right.$$
• **Vertical**: This filter simulates a vertical shadow. Thus, x_0_ and x_1_ are randomly selected in the interval [0, *h*[, and for each ***M****_ij_* the function *v(i, x_0_, x_1_)* as presented by Equation 2.
  (2) $$v\left(i,x_{0},x_{1}\right)=\left\{ \begin{array}{cc} 255 & if\;x_{0}\leq j\leq x_{1}\\ 0 & \text{otherwise} \end{array}\right.$$
• **Rectangle**: This filter simulates a combination of the horizontal and vertical shadows, where the points y0, y1, x0 and x1 are selected in the same manner as above. Thus, the combination is presented by Equation 3.
  (3) $$rect\left(i,j,x_{0},x_{1},y_{0},y_{1}\right)=h\left(j,y_{0},y_{1}\right)<>v\left(i,x_{0},x_{1}\right)$$

where <> is defined as per Table 2.

• **Tree**: This filter simulates shadows caused by trees. Therefore, *max_v_* is randomly selected in interval [0,999], and for each *M_ij_* the Eq. (4) is applied.
  (4) $$tree\left(i,j,max_{v}\right)=\left\{ \begin{array}{cc} 0 & if\;random\;x\;between\;\left[0,\max_{v}\right]=0\\ 255 & otherwise \end{array}\right.$$

**Table 2:** Definition of 〈〉.

*h*	*<>v*	*h<>v*
0	0	0
0	255	0
255	0	0
255	255	255

The second phase consists of acquiring the identification of the vehicle and detecting the boundary box of the license plate. This was achieved by using a Tiny-YOLOv3 model trained to detect legacy three-letter license plates under the neural network hyper-parameters and Darknet53 architecture configuration, summarized in Table 3. The Tiny-YOLOv3 setup adhered to the default CNN configuration [5] except the number of classes and filters were set to 1 and 18, respectively. Also, to enable the training process to be stopped and restored, the current weights were saved every 100 iterations. At the end of training the weights with the lowest average loss were selected.

**Table 3:** Tiny-YOLOv3 neural network configuration.

Params	Values
batch	24
subdivisions	8
width	416
height	416
channels	3
momentum	0.9
decay	0.0005
angle	0
saturation	1.5
exposure	1.5
hue	0.1
learning_rate	0.001
burn_in	400
max_batches	10,000
policy	steps
steps	3800
scales	0.1

**Fig. 3:**
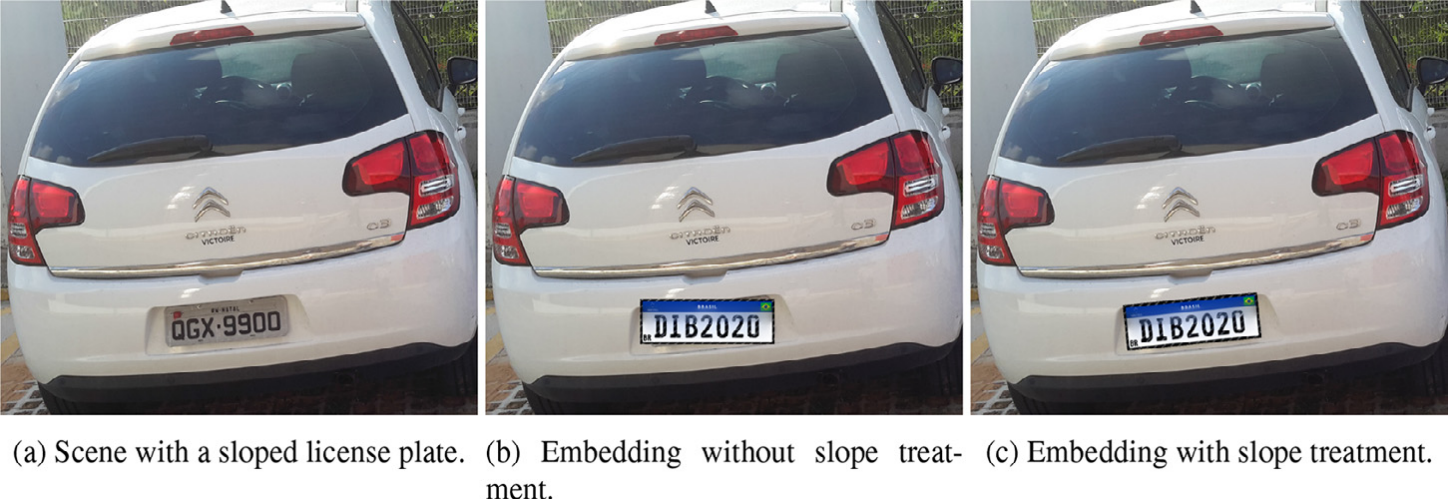
Scenario with inclined license plate.

Lastly, in the third phase, the information acquired in Phase 2 is used to complete the synthetic license plate and the boundary box is used to position it and overwrite the original license plate. The angle was obtained by a cut performed on the detected boundary box of the legacy three-letter license plate, and an adaptive threshold technique is applied to the sloped license plate using OpenCV, considering the mean of neighbourhood as divisor [4]. Then, the Probabilistic Hough Transformation is applied to detect lines [6] and its angles and mean are calculated. Finally, the newly generated license plate is placed in the scene at the correct position and orientation (Fig. 3) so that the slope is captured and the synthetic license plate replaces the original images as closely as possible.

## Declaration of Competing Interest

The authors declare that they have no known competing financial interests or personal relationships that have, or could be perceived to have, influenced the work reported in this article.
